# Isolation, characterization, and antimicrobial susceptibility pattern of *Escherichia coli* O157:H7 from foods of bovine origin in Mekelle, Tigray, Ethiopia

**DOI:** 10.3389/fvets.2022.924736

**Published:** 2022-11-17

**Authors:** Getachew Gugsa, Million Weldeselassie, Yisehak Tsegaye, Nesibu Awol, Ashwani Kumar, Meselu Ahmed, Nigus Abebe, Habtamu Taddele, Abrha Bsrat

**Affiliations:** ^1^School of Veterinary Medicine, Wollo University, Dessie, Ethiopia; ^2^Shire Agricultural Technical Vocational and Education Training College, Shire, Ethiopia; ^3^College of Veterinary Sciences, Mekelle University, Mekelle, Ethiopia; ^4^College of Agriculture and Veterinary Medicine, Addis Ababa University, Addis Ababa, Ethiopia

**Keywords:** antimicrobial, bacteriological, bovine, *E. coli* O157:H7, food, Mekelle, molecular

## Abstract

*Escherichia coli* O157:H7 is an emerging and major zoonotic foodborne pathogen. It has an increasing concern about the spread of antimicrobial-resistant strains. This study aimed to isolate and characterize Shiga toxin-producing *E. coli* O157:H7 from raw milk, yogurt, and meat of bovine origin and determine their antimicrobial susceptibility pattern. A cross-sectional study was conducted from December 2014 to June 2015, and a total of 284 milk and meat samples were collected from different sources in Mekelle. The collected samples were analyzed for the presence of *E. coli* and Shiga toxin-producing *E. coli* O157:H7 and the determination of their antimicrobial susceptibility pattern following the standard bacteriological and molecular techniques and procedures and antimicrobial sensitivity test. Out of the total 284 samples, 70 (24.6%) were bacteriologically positive for *E. coli* and 14.3% were found to be Shiga toxin-producing *E. coli* O157:H7. Of note, 100% of *E. coli* isolates carried the *pal* gene and 41.7% *eaeA* gene (EHEC). Of these EHEC isolates, 40% and 60% were positive for *stx1* and *stx2*, respectively. *E. coli* isolates showed the highest level of susceptibility to gentamycin (91.7%) but the highest level of resistance to amoxicillin (95.8%). Of the tested isolates, 18 (75%) of *E. coli* showed multidrug-resistant. This study revealed the occurrence of Shiga toxin-producing *E. coli* O157:H7 in foods of bovine origin in the study area. In conclusion, a nationwide phenotypic and molecular characterization, in-depth typing, and drug-resistant gene identification of *E. coli* O157:H7 should be undertaken.

## Introduction

In Ethiopia, both food shortage and lack of appropriate food safety assurance systems are problems that have become obstacles to the country's economic development and public health safety (1, 2). Animal products are generally regarded as high-risk commodities in respect of pathogen contents, natural toxins, and other possible contaminants and adulterants (3). Foodborne microorganisms are major pathogens affecting food safety and cause human illness worldwide as a result of the consumption of foodstuff, mainly animal products contaminated with vegetative pathogens or their toxins. Most of these microbes have zoonotic importance, resulting in a significant impact on both public health and economic sectors (4). Moreover, the emergence of multidrug-resistant (MDR) pathogens presents a global challenge for treating and preventing disease spread through zoonotic transmission (5).

Data regarding foodborne diseases are extremely scarce at a national level, and a few studies conducted in different parts of the country showed the poor sanitary conditions of catering establishments and the presence of pathogenic organisms such as *Campylobacter, Salmonella, Staphylococcus aureus, Bacillus cereus*, and *Escherichia coli* (6–10).

*Escherichia coli* found in humans can be categorized on basis of genetic and clinical criteria into the following three main groups: commensal, pathogenic (enteric or diarrheagenic), and extraintestinal pathogenic *E. coli* (ExPEC) (11). The typical diarrheagenic strains include enterotoxigenic (ETEC), enterohemorrhagic (EHEC), enteroinvasive (EIEC), enteropathogenic (EPEC), and enteroaggregative (EAEC) *E. coli* (12, 13). The *E. coli* that cause enteric disease have been divided into pathotypes, based on their virulence factors and mechanisms by which they cause disease (11, 14). One of these pathotypes, called Shiga toxin-producing *E. coli* (STEC), refers to those strains of *E. coli* that produce at least 1 member of a class of potent cytotoxins called Shiga toxin. The STEC is also called verotoxin-producing *E. coli* (VTEC). The names Shiga toxin (Stx), derived from similarity to a cytotoxin produced by *Shigella dysenteriae* serotype 1 (15), and verotoxin (VT), based on cytotoxicity for Vero cells (16), are used interchangeably. Those STEC that cause hemorrhagic colitis and hemolytic uremic syndrome are called enterohemorrhagic *E. coli* (EHEC) (14, 17). The Shiga toxin-producing *E. coli* O157 is synonymous with *E. coli* O157:H7 (18, 19). Pathogenicity of *E. coli* O157:H7 is encoded by a variety of plasmid, bacteriophage, and chromosomal genes (20). The key virulence factor for the subset of EHEC is the Shiga toxin (*Stx*) family, which contains two subgroups, namely, *Stx1* and *Stx2* that share approximately 55% amino acid homology (11). The ability to produce Shiga toxin was acquired from a bacteriophage presumably directly or indirectly from *Shigella* (20).

Shiga toxin-producing *E. coli* is recognized as an important cause of food-borne disease in humans and causes large outbreaks worldwide (21). *E. coli* O157:H7 is the leading cause of hemorrhagic colitis and hemolytic-uremic syndrome (HUS), a life-threatening sequela characterized by a triad of acute renal failure, microangiopathic hemolytic anemia, and thrombocytopenia in humans (22, 23). People of all ages are susceptible to infection with STEC. However, the young and the elderly are more susceptible and are more likely to develop more serious symptoms (24). Domestic and wild animals are sources of EHEC O157:H7 but the major animal carriers are healthy domesticated ruminants, primarily cattle, and to a lesser extent, sheep, and possibly goats. Fresh meat and raw milk are, nevertheless, considered common vehicles for *E. coli*, particularly for the EHEC (O157:H7) strain. Contamination of meat usually occurs during animal slaughter, as a result of poor slaughter practices, abattoir hygiene, and animal handling practices (20, 25).

Foods of bovine origin are frequently implicated in human outbreaks of Shiga toxin-producing *E. coli* (STEC) O157 (26, 27). Shiga-toxigenic *E. coli* is transmitted by the fecal-oral route by either consumption of contaminated food or water, from direct contact with infected animals, or *via* person-to-person contact. Moreover, a series of studies on the resistance of *E. coli* isolated from animals and humans have strongly suggested that those bacteria that are resistant to antimicrobials used in animals would also be resistant to antimicrobials used in humans (28–30). In general, antimicrobial resistance is a global public health problem, and growing scientific evidence indicates that it is negatively impacted by both human and animal antimicrobial usage (31). Although antimicrobial therapy is not the primary tool for treating infections caused by STEC O157:H7, MDR STEC O157:H7 is a public health issue as those strains participate in a reservoir of resistance genes that could be easily exchanged between Enterobacteriaceae in the host and the environment (32). However, less is currently known about the molecular basis of MDR in STEC O157:H7 isolates of food origin (33). Therefore, it is of paramount importance to systematically investigate and characterize this recurring food-borne disease. Thus, the current study was conducted to isolate and characterize Shiga toxin-producing *E. coli* O157:H7 from raw milk, yogurt, and meat of bovine origin from different sources in the study area and to determine the antimicrobial susceptibility pattern of the isolates.

## Materials and methods

### Study area

This study was conducted from December 2014 to June 2015 in Mekelle City. Mekelle is the capital city of Tigray Regional State, situated approximately 783 km North of Addis Ababa at 38.5° East longitude and 13.5° North latitude at an altitude of 2,300 above sea level. The city has a total population of 215,546 (34), 308 cafeterias, 292 restaurants, 258 supermarkets, and an active urban-rural exchange of goods which has 30,000 micro and small enterprises (35). The weather condition is hot and dry. The mean annual rainfall of the area is 628.8 mm and an annual average temperature of 21°C (36).

### Study design and study population

A cross-sectional study on Shiga toxin-producing *E. coli* O157:H7 was conducted from December 2014 to June 2015 on raw milk, yogurt, and meat samples collected from different sources and parts of Mekelle City, Tigray, Ethiopia. The study populations comprised purposively selected milking dairy cows and slaughtered cattle found in Mekelle City.

### Sampling technique and sample collection

A total of 284 samples of bovine origin, comprised of raw milk (*n* = 145), yogurt (*n* = 48), and meat (*n* = 91), were collected using a purposive random sampling technique. These samples were collected based on the willingness of the owners until the required sample size was achieved. Raw milk samples were aseptically collected directly from the teats of lactating cows (*n* = 100), whole-sellers (*n* = 17), cafeterias (*n* = 28), and similarly the yogurt samples were collected from dairy farms (*n* = 26) and cafeterias (*n* = 22) using a sterile universal bottle. However, the raw meat samples were collected from abattoirs (*n* = 55), butchery shops (*n* = 16), and restaurants (*n* = 20) during slaughtering and selling. Then, the sections of meat samples were aseptically removed and placed in separate sterile plastic bags to prevent spilling and cross-contamination. Both samples were transported to the Veterinary Microbiology Laboratory of the College of Veterinary Sciences, Mekelle University using an icebox and stored at +4°C until the laboratory work was conducted.

### Isolation and identification of *E. coli* and *E. coli* O157:H7

#### Microbiological procedures

Isolation of *E. coli* was attempted according to Quinn et al. (37) with slight modification. A part of each sample (10 ml or 10 g) was enriched in peptone water (HiMedia, Mumbai, India) (90 ml) and was incubated at 37°C for 24 h. Enriched samples were inoculated on MacConkey Agar (MCA) (HiMedia, Mumbai, India) by four flame techniques, and plates were incubated at 37°C for 24 h. Pink-colored colonies were considered presumptive of *E. coli*. Gram staining was performed as per procedures described by Merchant and Packer (38) to determine the size, shape, and arrangement of bacteria. The organisms revealed Gram-negative, pink-colored with rod-shaped appearance, and arranged in single or in pairs were suspected as *E. coli*.

A single well-isolated colony was picked from MCA and streaked on Eosin Methylene Blue Agar (EMB) (HiMedia, Mumbai, India) and incubated at 37°C for 24 h. The characteristic green metallic sheen growth of colonies is a presumptive identification for *E. coli*. Colony morphology and colors on MCA and EMB agar plates together with the Gram-stain procedure were used as an initial identification of *E. coli* colonies (39). Such colonies were taken from EMB into nutrient broth and agar for further biochemical examination. Standard biochemical tests (catalase test, indole, methyl red, Voges-Proskauer test, nitrate reduction, citrate utilization, and urease production) were used as confirmation of identification (40–46). The Triple Sugar Iron test was performed according to Vanderzant and Splittstresser (47). Carbohydrate fermentation tests of the isolates were performed according to the method of Simmons (43). Colonies that were confirmed as *E*. coli were further subcultured onto MacConkey Agar with Sorbitol to differentiate *E. coli* O157: H7 from other strains, especially lactose fermenters *E. coli*. A bacterial strain that was used as a quality control organism in this study was a standard strain of *E. coli* ATCC 25922.

#### PCR amplification of the pal, eaeA stx1, and sxt2 virulence genes of E. coli

*Escherichia coli* genomic DNA extraction and purification were performed as per the protocol given by PureLink^®^ Genomic DNA Purification Kit, USA, for Gram-negative organisms, and the total genomic DNA was checked by running on 1.0% agarose gel.

The DNA of all presumptive isolates was subjected to multiplex PCR for analyzing the presence of the *pal* (48), *eaeA* (49)*, stx1*, and *stx2* (50) genes and further modified by Fitzmaurice (51). The amplification was performed according to the protocol reported by Parekh and Subhash (52) and Wang et al. (53) using the following specific primers: ECPAL-F-5′GGC AAT TGC GGC ATG TTC TTC C3′ and ECPAL-R-5′CCG CGT GAC CTT CTA CGG TGA3′ for *pal* gene of *E. coli* (280 bp); EHEC-F-5′TGG TAC GGG TAA TGA AAA3′ and EHEC-R-5′AAT AGC CTG GTA GTC TTG T3′ for *eaeA* gene of EHEC (360 bp); *Stx1*-F-5′ATA AAT CGC CAT TCG TTG ACT AC3′ and *Stx1*-R-5′AGA ACG CCC ACT GAG ATC ATC3′ for *stx1* (180 bp); and *Stx2*-F-5′GGC ACT GTC TGA AAC TGC TCC3′ and *Stx2*-R-5′TCG CCA GTT ATC TGA CAT TCT G3′ for *stx2* (255 bp). Each reaction mixture (50 μl) consisted of 5 μl of 10× reaction buffer (500 mM KCl, 15 mM MgCl_2_, 100 mM Tris HCl pH 8.3, 0.1%w/v gelatin), 5 μl of template DNA, 1 μl of each primer (the primers were used at a final concentration of 100 M), 3 μl of 10 mM dNTP mixture (at a concentration of 100 μl each), and 1 μl of Taq polymerase (3 U/l). The remaining volume of the reaction mixture was nuclease-free water. The amplification was carried out using a Tianlong PCR Thermocycler with thermal cycling conditions of an initial denaturation at 94°C for 6 min, followed by 35 cycles of denaturation at 94°C for 45 s, annealing at 55°C for 30 s, extension at 72°C for 45 s, and with a final extension at 72°C for 6 min. Finally, PCR products were separated in a horizontal equipment system by running on a 1.5% (w/v) agarose gel containing 0.5 g/ml ethidium bromide for 55 min at 110 V using 1XTAE buffer (40 mM Tris, 1 mM EDTA, and 20 mM glacial acetic acid, pH 8.0). The amplicons were visualized under UV-light gel doc, and their molecular weight was estimated by comparing them with a 100 bp DNA molecular weight marker (Solis BioDyne, Tartu, Estonia) (54).

#### Antibiotic susceptibility testing

The isolates of *E. coli* were screened for *in vitro* antimicrobial susceptibility using the agar disk diffusion method described by Bauer et al. (55). For this, the following seven different antibiotic disks (Oxoid Ltd., Basingstoke, Hampshire, England) with their concentrations given in parentheses were used in the antibiograms: amoxicillin (AMX) (2 μg), doxycycline (DOX) (30 μg), erythromycin (ERY) (15 μg), gentamicin (GEN) (10 μg), penicillin (PEN) (10 μg), trimethoprim-sulfamethoxazole (SXT) (20 μg), and tetracycline (TET) (30 μg). The selection of these antibiotics was based on the availability and frequent use of these antimicrobials in the study area both in veterinary and human medicine. Four to five well-isolated colonies from nutrient agar plates were transferred into tubes containing 5 ml of a normal saline solution until it achieved the 0.5 McFarland turbidity standards, and then a sterile cotton swab was dipped into the adjusted suspension and then spread evenly over the entire surface of the plate of Mueller-Hinton agar (Oxoid Ltd., Basingstoke, Hampshire, England) to obtain uniform inoculums. The plates were then allowed to dry for 3–5 min. Antibiotic-impregnated disks were then applied to the surface of the inoculated plates with sterile forceps. After 18–24 h of incubation at 37°C, the plates were examined, and the clear zone (inhibition zones of bacterial growth around the antibiotic disk including the disk) diameter for individual antimicrobial agents was measured using a digital caliper and then translated into susceptible (S), intermediate (I), and resistant (R) categories according to the interpretation table of the Clinical and Laboratory Standard Institute (56).

#### Data management and analysis

All collected data were entered into Microsoft Excel Sheet and analyzed through the SPSS version 16. Accordingly, descriptive statistics such as percentages and frequency distribution were used to determine the occurrence of the food items, and the chi-square (χ^2^) test was applied to assess the association.

## Results

### The occurrence of *E. coli* and *E. coli* O157:H7

Out of the total 284 samples collected from the different sample sources, 70 (24.6%) were found to be positive for *E. coli*. The sample-based positivity of meat, raw milk, and yogurt samples were 41 (45.1%), 18 (12.4%), and 11 (22.9%), respectively. There was a significant difference (χ^2^ = 32.1; *p* = 0.000) among the different sample types in the occurrence of *E. coli* (Table 1). Of the total isolated *E. coli* isolates, 14.29% were found to be Shiga toxin-producing *E. coli* O157:H7.

**Table 1:** Occurrence of *Escherichia coli* among the different sample types.

**Sample types**	**No. of examined**	**No. (%) of positive samples**	**χ^2^**	***P-value***
Meat	91	41 (45.1%)		
Raw milk	145	18 (12.4%)	32.1	0.000
Yogurt	48	11 (22.9%)		
Total	284	70 (24.6%)		

Occurrence rates of 56.3%, 40%, and 50% were recorded in meat samples collected from butchery, abattoir, and restaurant, respectively. The positivity of *E. coli* for raw milk samples collected from farms, whole-sellers, and cafeterias were 10, 17.6, and 17.9%, respectively, whereas yogurt samples collected from farms and cafeterias were found to be 23.1 and 22.7%, respectively. The chi-square analysis result revealed that all sample collection sources showed a significant difference (χ^2^= 35.2; *p* = 0.000) in *E. coli* positivity (Table 2).

**Table 2:** Occurrence of *E. coli* among the different sample types and sample sources.

**Sample type**	**Site of collection**	**Total No. of samples examined**	**Total No. of samples positive**	**χ^2^**	***P-value***
Meat	Abattoir	55	22 (40%)		
	Butchery	16	9 (56.3%)	35.2	0.000
	Restaurant	20	10 (50%)		
	Total	91	41 (45.1%)		
Raw milk	Farm	100	10 (10%)		
	Whole seller	17	3 (17.6-%)	35.2	0.000
	Cafeteria	28	5 (17.9%)		
	Total	145	18 (12.4%)		
Yogurt	Farm	26	6 (23.1%)		
	Cafeteria	22	5 (22.7%)	35.2	0.000
	Total	48	11 (22.9%)		

### PCR amplification results of the virulence genes of *E. coli*

The PCR result indicated that all (100%) of the tested isolates of *E. coli* carried the *pal gene* and 41.67% *eaeA gene* (EHEC). Among the *eaeA* gene-carried *E. coli* isolates, 40% carried the *stx1* gene and 60% of them carried the *stx2* gene.

### Antimicrobial susceptibility profile of *E. coli* isolates

The *E. coli* strains isolated from meat, raw milk, and yogurt showed the highest level of susceptibility to gentamycin (91.7%). However, the highest level of resistance was observed against amoxicillin (95.8%) (Table 3). MDR was detected in 75% of the isolates.

**Table 3:** *In vitro* antimicrobial drug susceptibility results of *E. coli* isolates.

**Antimicrobial agents**	**Results**		
	**Sensitive**	**Intermediate**	**Resistant**
Gentamycin	22 (91.7%)	0%	2 (8.3%)
Penicillin	1 (4.2%)	1 (4.2%)	22 (91.7)
Amoxicillin	1 (4.2%)	0%	23 (95.8%)
Doxycycline	10 (41.7%)	2 (8.3%)	12 (50%)
Tetracycline	14 (58.3)	0%	10 (41.7%)
Sulfamethoxazole	19 (79.2%)	0%	5 (20.8%)
Erythromycin	7 (29.2)	4 (16.7%)	13 (54.2%)

## Discussion

This study revealed an overall positivity rate of 24.6% for *E. coli*. This finding was in line with the reports of Bedasa et al. (57) (20%), Sebsibe and Asfaw (58) (20.2%), Messele et al. (59) (21.6%), Tadese et al. (60) (23.4%), Abebe et al. (61) (23.7%), Haileselassie et al. (10) (27.3%), and Haftu et al. (62) (27.3%). It was higher than the reports of Kumar et al. (63) (8.15%), Yakubu et al. (64) (9.23%), Atnafie et al. (65) (12.38%), Mohammed et al. (66) (15.89%), and Ababu et al. (67) (19%). However, it was lower than the findings of Disassa et al. (68) (33.9%), Thaker et al. (69) (38%), Negesse et al. (70) (46.26%), Balcha et al. (71) (61.7%), and Ali et al. (72) (63%). This study also revealed an overall positivity of 14.3% for Shiga toxin-producing *E. coli* O157:H7 (EHEC). This finding was almost in agreement with the reports of Bekele et al. (73) (10.2%), Abebe et al. (61) (10.4%), Mekuria and Beyene (74) (10.4%), Akomoneh et al. (75) (10.9%), Beyi et al. (76) (11.43%), Mohammadi et al. (77) (17.47%), and Balcha et al. (71) (18%). However, it was higher than the findings of Abdissa et al. (78) (0.54%), Yakubu et al. (64) (1.92%), Geresu and Regassa (79) (2.1%), Mohammed et al. (65) (2.4%), Carney et al. (80) (2.4%), Disassa et al. (68) (2.9%), Ahmed and Shimamoto (81) (3.38%), Bedasa et al. (57) (3.5%), Hiko et al. (82) (4.2%), Ababu et al. (67) (5.2%), Sebsibe and Asfaw (58) (5.4%), Gutema et al. (83) (6.3%), Vanitha et al. (84) (8.8%), Dutta et al. (85) (9%), Tadese et al. (60) (9.1%), and Haile et al. (86) (9.3%). However, it was lower than the finding of Garbaj et al. (87) (25%), Llorente et al. (88) (36.1%), Nehoya et al. (89) (41.66%), and Islam et al. (90) (69%). The variation might be due to differences in sample types of food of bovine origin, sources of the samples, methodological approaches, diagnostic techniques, calculation/interpretation, geographical locations, hygienic conditions, handling and transportation of samples, and contamination rates from utensils and personnel. Generally, in the present findings, the positivity of *E. coli* was higher in the samples collected from butchery, restaurants, cafeterias, and whole sellers other than the other sources. This might be due to food establishments in the study area being found to have poor sanitation and were not maintained well, specifically, poor repair condition of kitchens, improperly managed toilet facilities, inappropriate solid waste receptacles, lack of standard dishwashing compartments, and lack of handwashing lavatories in a large number of establishments (8).

The antimicrobial susceptibility test results of *E. coli* strains isolated from meat, raw milk, and yogurt showed the highest level of susceptibility for gentamycin (91.7%). It was almost in agreement with the reports of Ababu et al. (67) (100%), Bedasa et al. (57) (82.5%), and Hailu (91) (81.82%). The 79.2% isolates were susceptible to trimethoprim-sulfamethoxazole, which was in agreement with the reports of Hailu (91) (78.79%). However, the highest level of resistance was observed against amoxicillin (95.8%), which was in line with the reports of Tadese et al. (60) (100%) and Abdissa et al. (78) (100%). In addition, 41.7% of the isolates were resistant to tetracycline, which was in agreement with the reports of Messele et al. (59) (47.6%) but was higher than the findings of Carvalho et al. (92) (4%), Bekele et al. (73) (5.1%), Mohammadi et al. (77) (23.08%), Tadese et al. (60) (27.7%), and Mora et al. (93) (32%). However, it was lower than the findings of Ahmed and Shimamoto (81) (80.6%), Disassa et al. (68) (81.8%), Ababu et al. (67) (63.63%), and Hailu (91) (60.61%). This study also revealed that 20.8% of the isolates showed resistance against Sulfamethoxazole, which was lower than the findings of Mora et al. (93) (36%). In general, in this study, 75% of the isolates developed MDR, which was higher than the reports of Messele et al. (59) (46.0%) and Tadese et al. (60) (66.3%) but was lower than the report of Mekuria and Beyene (74) (93.2%). Antimicrobial resistance may arise either spontaneously by selective pressure or due to antimicrobial misuse by humans or overuse in the feeding or treatment of animals by farmers (94).

## Conclusion

This study revealed a relatively high occurrence of *E. coli* as well as Shiga toxin-producing *E. coli* O157:H7 in food of bovine origin in Mekelle City and isolates developed a high level of MDR (a bacterium that is resistant to more than one antibiotic) to the antimicrobials tested. Hence, foods of bovine origin can serve as a potential vehicle for transmitting *E. coli*, in particular, Shiga toxin-producing *E. coli* O157:H7, and its presence indicates a serious public health hazard and gives a warning signal for the possible occurrence of a foodborne outbreak in humans through the consumption of raw or undercooked food items. As a result, both its presence and development of MDR should receive particular attention and is an alert for the concerned bodies. Therefore, a coordinated effort is needed to reduce or eliminate the risk posed by these pathogens at various stages in the food chains and on the appropriate use of antimicrobials both in veterinary and human treatment regimes. Moreover, awareness creation should be made on foodborne diseases caused by Shiga toxin-producing *E. coli* O157:H7 with due consideration to the safe handling and consumption of food of animal origin and selection and safe use of antimicrobials. In conclusion, a nationwide phenotypic and molecular characterization, in-depth typing, and drug-resistant gene identification of *E. coli* O157:H7 should be undertaken.

## Data availability statement

The original contributions presented in the study are included in the article/supplementary material, further inquiries can be directed to the corresponding author.

## Ethics statement

The animal study was reviewed and approved by Institutional Ethical and Environmental Considerations Review Committee, College of Veterinary Sciences, Mekelle University. Written informed consent was obtained from the owners for the participation of their animals in this study.

## Author contributions

GG, MW, YT, and NeA participated in conception and proposal development, sample collection, laboratory analysis, supervision, validation, data analysis, and write-up of the original draft and reviewing and editing of the final version of the manuscript. AK, MA, NiA, HT, and AB were involved in the conception and proposal development, data analysis, validation, write-up of the original draft, and reviewing and editing of the final version of the manuscript. All authors contributed to the final version of the manuscript and approved the submission.

## Funding

This study was funded by the College of Veterinary Sciences, Mekelle University.

## Conflict of interest

The authors declare that the research was conducted in the absence of any commercial or financial relationships that could be construed as a potential conflict of interest.

## Publisher's note

All claims expressed in this article are solely those of the authors and do not necessarily represent those of their affiliated organizations, or those of the publisher, the editors and the reviewers. Any product that may be evaluated in this article, or claim that may be made by its manufacturer, is not guaranteed or endorsed by the publisher.

## Abbreviations

DNA: deoxyribonucleic acid
*E. coli* O157: *Escherichia coli* O157
*E. coli*: *Escherichia coli*
EAEC: Enteroaggregative *E. coli*
EHEC: Enterohemorrhagic *E. coli*
EIEC: Enteroinvasive *E. coli*
EPEC: Enteropathogenic *E. coli*
ETEC: Enterotoxigenic *E. coli*
ExPEC: Extraintestinal pathogenic *E. coli*
HUS: hemolytic-uremic syndrome
MDR: multidrug-resistant
PCR: polymerase chain reaction
STEC: Shiga toxin-producing *E. coli*
Stx: Shiga toxin
TTP: thrombotic thrombocytopenic purpura
VT: verotoxin
VTEC: verotoxin-producing *E. coli*.
